# Isolation identification and biochemical characterization of a novel halo-tolerant lipase from the metagenome of the marine sponge *Haliclona simulans*

**DOI:** 10.1186/1475-2859-11-72

**Published:** 2012-06-01

**Authors:** Joseph Selvin, Jonathan Kennedy, David PH Lejon, G Seghal Kiran, Alan DW Dobson

**Affiliations:** 1 Microbial Genomics Research Unit, Department of Bioinformatics, Bharathidasan University, Tiruchirapalli, India; 2 Marine Biotechnology Centre, Environmental Research Institute, University College Cork, Cork, Ireland; 3 Department of Microbiology, University College Cork, Cork, Ireland; 4 Laboratoire d’étude des Transferts en Hydrologie et Environnement, UMR 5564 (CNRS/INPG/IRD/UJF), Université de Grenoble, Grenoble, France; 5 Department of Microbiology, Pondicherry University, Puducherry, India

**Keywords:** Metagenomic library, Functional screening, Lipase, Marine sponge

## Abstract

**Background:**

Lipases (EC 3.1.1.3) catalyze the hydrolysis of triacyl glycerol to glycerol and are involved in the synthesis of both short chain and long chain acylglycerols. They are widely used industrially in various applications, such as baking, laundry detergents and as biocatalysts in alternative energy strategies. Marine ecosystems are known to represent a large reservoir of biodiversity with respect to industrially useful enzymes. However the vast majority of microorganisms within these ecosystems are not readily culturable. Functional metagenomic based approaches provide a solution to this problem by facilitating the identification of novel enzymes such as the halo-tolerant lipase identified in this study from a marine sponge metagenome.

**Results:**

A metagenomic library was constructed from the marine sponge *Haliclona simulans* in the pCC1fos vector, containing approximately 48,000 fosmid clones. High throughput plate screening on 1% tributyrin agar resulted in the identification of 58 positive lipase clones. Following sequence analysis of the 10 most highly active fosmid clones the pCC1fos53E1 clone was found to contain a putative lipase gene *lpc*53E1, encoded by 387 amino acids and with a predicted molecular mass of 41.87 kDa. Sequence analysis of the predicted amino acid sequence of Lpc53E1 revealed that it is a member of the group VIII family of lipases possessing the SXTK motif, related to type C β-lactamases. Heterologous expression of *lpc*53E1 in *E. coli* and the subsequent biochemical characterization of the recombinant protein, showed an enzyme with the highest substrate specificity for long chain fatty acyl esters. Optimal activity was observed with *p*- nitrophenyl palmitate (C_16_) at 40°C, in the presence of 5 M NaCl at pH 7; while in addition the recombinant enzyme displayed activity across broad pH (3–12) and temperature (4 -60°C) ranges and high levels of stability in the presence of various solvents at NaCl concentrations as high as 5 M and at temperatures ranging from 10 to 80°C. A maximum lipase activity of 2,700 U/mg was observed with 10 mM *p*-nitrophenyl palmitate as substrate, in the presence of 5 mM Ca^2+^ and 5 M NaCl, and a reaction time of 15 min at pH 7 and 40°C; while K_M_ and *Vmax* values were calculated to be 1.093 mM^-1^ and 50 μmol/min, respectively.

**Conclusion:**

We have isolated a novel halo tolerant lipase following a functional screen of a marine sponge fosmid metagenomic library. The activity and stability profile of the recombinant enzyme over a wide range of salinity, pH and temperature; and in the presence of organic solvent and metal ions suggests a utility for this enzyme in a variety of industrial applications.

## Background

Marine ecosystems represent a large and as yet largely under explored reservoir of biodiversity with respect to industrially useful biocatalysts. These ecosystems can range from coastal environments to deep-sea hydrothermal vents with high hydrostatic pressure and temperatures as high as approximately 400°C. Marine ecosystems are also subject to low temperatures with the average temperature of the oceans being around 3°C. These diverse marine ecosystems clearly impose a number of constraints on the cellular processes of the microorganisms living and surviving within them [1]. Survival under these conditions must have necessitated the development of quite novel microbial cellular biochemistry and metabolism, thereby ensuring that these bacteria are likely to possess unique enzyme systems such as increased salt tolerance and cold adaptivity which match many industrial requirements [2].

Marine sponges (phylum: *Porifera*) are one such unique environmental niche, with sponges playing host to numerically vast and phylogenetically diverse bacterial communities [3]. These microbes, which can include bacteria, archaea and single celled eukaryotes (fungi and microalgae), may be symbiotic, pathogenic, a food source or in some instances simply be transiently associated; but in some sponges, up to 40% of the total biomass can comprise endosymbiotic microorganisms [4]. Enzymes produced by sponge associated microbes are likely to have a range of quite diverse biochemical and physiological characteristics that have permitted the microbial communities to adapt and ultimately thrive in these unique ecosystems. However the potential exploitation of these enzymes is somewhat hindered by the fact that the majority of the microbial species which are present within the sponge ecosystem are currently not amenable to culturing [5]. Indeed it is generally believed that only between 0.001 to 0.1% of marine microorganisms are readily culturable using currently available culturing approaches [6]. Metagenomic based approaches are currently being employed to overcome this particular bottleneck and thereby gain access to this untapped reservoir of novel enzymes [7,8].

Metagenomics involves harvesting bulk DNA from environmental samples (or from enrichment cultures), archiving it in libraries with appropriate heterologous hosts and either subsequently screening these libraries for a gene of interest using an homology based approach; or expression of the DNA and screening for enzymatic activities of interest [9,10]. Alternatively, these libraries may be subjected to high throughput shotgun sequencing and automated annotation of sequenced data [11].

Lipolytic enzymes (Esterases (EC 3.1.1.1) and Lipases (EC 3.1.1.3)) are one of the most important groups of biocatalysts that carry out novel reactions in both aqueous and non aqueous media. There is much current interest in lipases due to their potential utility in numerous industrial applications ranging from biodiesel production, to food flavoring, in laundry applications, cosmetic production, and in the paper and pharmaceutical industries [12]. They currently constitute the third largest enzyme group, after proteases and carbohydrases, with respect to overall market value. Despite the intense global interest in lipases, marine microbial lipases remain as yet quite unexploited.

Lipases are catalytic triad serine hydrolases, which catalyze the hydrolysis of triacyl glycerol to glycerol and the synthesis of both short chain (≤10) and long chain (≥10) acylglycerols [13]. Despite differences in size, sequence homology, substrates, activators, inhibitors, and other properties, most lipases adopt a similar core topology, known as the α β hydrolase fold. The interior topology of α β hydrolase fold proteins largely comprises of parallel β-pleated strands. There are at least five parallel β-pleated strands in lipases, which are typically separated by stretches of α-helix; forming an overall super helically twisted–pleated sheet [13].

Lipases differ from one another by size, substrate specificity, stability profile, and activity in the presence of various activators and inhibitors. Given the importance of lipases in various industrial applications, there is much interest in isolating novel enzymes from unique environmental niches. In this respect functional metagenomic based approaches have proven useful in the identification of lipases from metagenomes from various environments, with new families being proposed [14,15]. A number of lipolytic genes have also been cloned from marine sources, with novel lipases being identified from the microbiota of the sponges *Aplysina aerophoba*, *Hyrtios erecta* and *Cymbastela*[16-18]. Recently fifteen different lipolytic genes, encoding proteins of between 32 and 68% amino acid identity with existing proteins in the database have been reported from a metagenomic library constructed from South China Sea marine sediment [19].

In this study, a fosmid metagenomic library was constructed from the marine sponge *Haliclona simulans* and a positive clone pCC1Fos53E1 obtained by screening on LB agar containing 1% tributyrin. The putative lipase gene *lpc*53E1 which encodes a 387 amino acid protein which when heterologously expressed in *E.coli* DH5α cells was confirmed to possess lipolytic activity. The heterologously expressed recombinant lipase was characterized and optimum activity conditions were determined using the response surface method (RSM). The Lpc53E1 protein was characterized as a novel halotolerant lipolytic enzyme, with specificity for long-chain fatty acyl esters containing the active site motif SXTK, common to lipase family VIII and class C β- lactamases.

## Results

### Metagenomic library construction and screening for lipase clones

**Figure 1:**
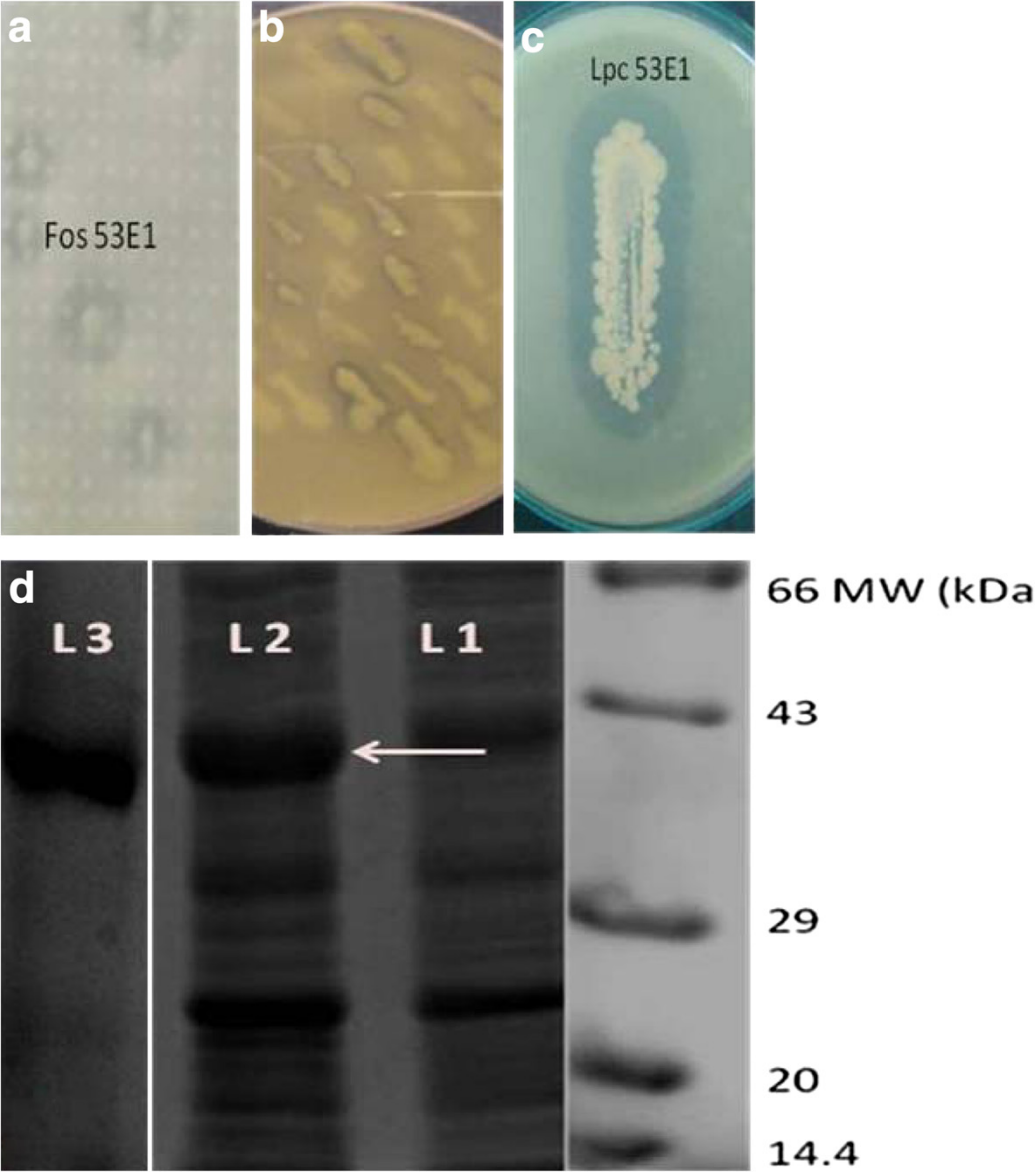
**Metagenomic screening, identification and purification of Lpc53E1.a)** Identification of clone 53E1 on 1% tributyrin agar, **b)** Lipase activity of *E. coli* clones carrying *lpc53E1*gene on plasmid, **c)** Lipolytic activity of *lpc53E1* clone on tributyrin agar plate. **d)** SDS–PAGE gel showing expression of HIS tagged Lpc53E1 lipase protein (indicated by arrow) in *E. coli* carrying *lpc53E1*gene on pBAD/Myc-HisA; L1 – crude extract of uninduced cells; L2- crude extract of induced cells; and L3 - Ni NTA column purified lipase.

A metagenomic library was constructed from the marine sponge *Haliclona simulans* as previously described [20]. The sponge *H. simulans* had been collected by SCUBA diving off the west coast of Ireland. Metagenomic DNA was extracted and size selected following pulse-field gel electrophoresis, electroelution of ~40 kb size DNA fragments and subsequently concentrated using an Amicon centrifugal concentrator. The library which was constructed using the fosmid vector pCC1FOS contained approximately 48,000 clones which were screened for lipase activity (Figure 1a). High throughput plate screening using 1% tributyrin resulted in the initial identification of 58 positive clones (data not shown). Among the 10 most highly active clones, the clone Lpc53E1 was subsequently sequenced.

### Pyrosequencing and CAMERA analysis

Fosmid DNA sequences were analyzed using the RAMMCAP pipeline hosted by CAMERA. A contig (contig 0004) derived from pCC1fos53E1 was identified as containing an ORF encoding a gene with potential lipase activity. The putative lipase ORF, named *lpc*53E1, was identified as a class C β-lactamase gene (COG1680 and Pfam00144 annotations). Blast analysis indicated that contig 0004 also contained additional predicted ORFs apparently unrelated to the predicted lipase activity (Genbank accession number JQ659262). Contig 0004 has a G-C content of 61.7% with lipase ORF *lpc*53E1 being 1164 bp, encoding a predicted protein of 387 amino acids.

### Cloning, expression and purification of recombinant Lpc53E1 lipase

The putative *lpc*53E1 lipase gene was PCR amplified, cloned into the pBAD/mycHis vector, transformed into *E. coli* DH5α and transformants were tested for lipolytic activity (Figure 1b and c). The over-expressed myc His-tagged lipase was subsequently purified using a ProBond™ Column. Active fractions were collected and pooled, and a lipase activity of 1900 U/mg was determined with *p*- nitrophenyl palmitate (C_16_) as substrate. Active fractions were further concentrated, dialyzed and analysed by SDS –PAGE. A lipase protein band consistent with the predicted mass of 44.76 kDa for the tagged lipase was observed in induced cells, which was absent in uninduced cells (Figure 1d). The molecular weight of Lpc53E1 was subsequently confirmed by MALDI- ToF analysis (data not shown).

### Lipase sequence analysis and phylogenetic tree construction

**Figure 2:**
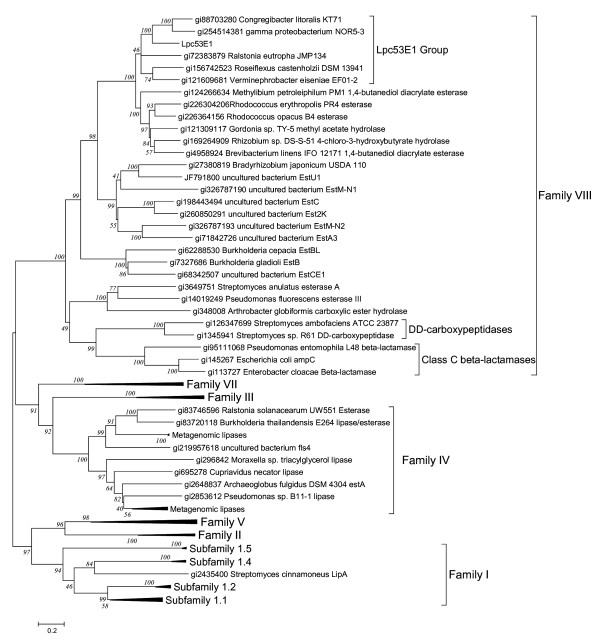
**Phylogenetic analysis of Lpc53E1 and closely related and other representative members of lipase families**. Lpc53E1 is a member of the Family VIII lipases and is a member of a group otherwise consisting of proteins of unknown function, putatively annotated as beta-lactamases.

**Figure 3:**
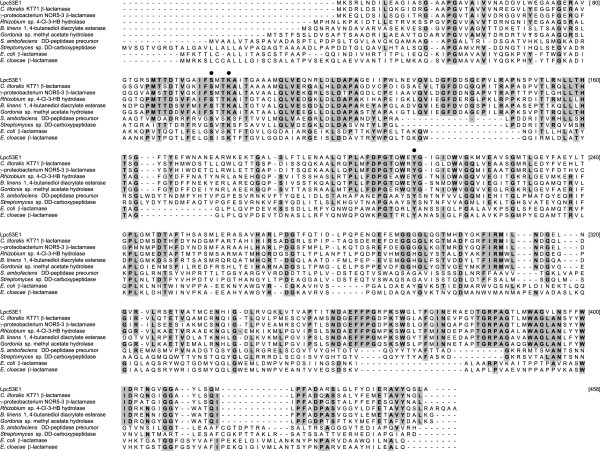
**Alignment of Lpc53E1 with related members of group VIII lipases**. The alignment includes; closely related putative β-lactamase proteins from *C. litoralis* and γ-proteobacterium NOR5; esterase and hydrolase enzymes from *Gordonia* and *B. linens*; dd-carboxypeptidases from *Streptomyces* sp., and; true class C β-lactamase enzymes from *E. coli* and *E. cloacae*. Amino acids shared by 6 or more proteins are shaded. The β-lactamase active site S, K and Y are indicated with ●. Alignment was performed with ClustalW using MEGA 5.

The Lpc53E1 lipase contained 387 amino acids with a predicted molecular weight of 41.87 kDa and a pI value of 4.61; and is a stable protein according to the instability index which is calculated to be 31.13. The predicted amino acid sequence of the Lpc53E1 protein displayed highest identity, of up to 59%, to several proteins classified as putative β-lactamases (*e.g.* β-lactamases from *Congregibacter litoralis* KT71 and the *γ* proteobacterium NOR5-3). However further analysis of all closely matching β-lactamase proteins indicated that these were annotated on the basis of sequence homologies with no accompanying biochemical or genetic evidence. Matching proteins with biochemical or genetic evidence of function included several hydrolytic enzymes including 4-chloro-3-hydroxybutyrate hydrolase (*Rhizobium* sp. DS-S-51), methyl acetate hydrolase (*Gordonia* sp. TY-5) and 1,4 butanediol diacrylate esterase (*Brevibacterium linens* IFO12171) (Figures 2 and 3). Alignment of the protein sequence of Lpc53E1 with known lipase families (Figure 2) showed that Lpc53E1 is a member of the family VIII esterase/lipases, a family of lipases that includes several enzymes isolated from metagenomic sources. However Lpc53E1 forms a separate group within the family VIII lipase clustering with proteins classified as group C β-lactamases.

Figure 3 shows a multiple sequence alignment between Lpc53E1 and other members of the group VIII lipases including type C β-lactamases. Analysis of this alignment shows that Lpc53E1 contains the S-X-X-K motif (residues 96–99 on Figure 3) that is conserved in family VIII carboxylesterases [13] and class C β-lactamases [21,22] and is conserved together with the active site tyrosine (residue 211). The G-x-S-x-G motif common to some lipase protein families and present in some members of the groupVIII lipases is absent from Lpc53E1 and closely related proteins.

### Biochemical characterization of recombinant lipase Lpc53E1

#### Substrate specificity of Lpc53E1

 The substrate specificity of the purified Lpc53E1 protein was assessed. Lpc53E1 displayed the highest activity with long-chain fatty acyl esters such as *p*-nitrophenyl palmitate (C_16_) (*p*NPP), *p*-nitrophenyl myristate (C_14_) (*p*NPM) and *p*-nitrophenyl laurate (C_12_) (*p*NPL); with *p*NPP resulting in a specific activity of 1,900 U/mg protein (pH7 and 40°C). This was subsequently taken to represent 100% relative activity (Table 1). Lower levels of activity were observed with shorter chain fatty acyl esters such as *p*-nitrophenyl caprylate (C8) (*p*NPC), *p*-nitrophenyl butyrate (C4) (*p*NPB) with *p* -nitrophenyl acetate (C2) (*p*NPA) displaying the lowest overall relative activity of approximately 23%. The K_M_ and *Vmax* values of lipase enzyme were found to be 1.093 mM^-1^ and 50 μmol/min, respectively, at standard assay condition with pNPP as substrate (Additional file 1). Purified Lpc53E1 was also assayed for β-lactamase activity against penicillin G, ampicillin and cephalexin, no β-lactamase activity was detected (data not shown).

**Table 1:** Effects of physiochemical parameters on Lpc53E1 activity: substrate specificity, temperature, pH, metals, NaCl concentrations, detergents & others

***Variables***	***Relative activity (%) (SD(yEr-))***	***Variables***	***Relative activity (%) (SD(yEr-))***	***Variables***	***Relative activity (%) (SD(yEr-))***	***Variables***	***Relative activity (%) (SD(yEr-))***
**Substrate specificity**		**Metals 1mM**		**Metals 10 mM**		**NaCl**	
*p*NPP	100.0 ± 0.7	Control	100.0 ± 0.1	Control	100.0 ± 0.1	0M	100 ± 0.08
*p*NPM	88.3± 2.4	Ag^+^	132.3 ± 3.5	Ag^+^	38.2 ± 2.5	1M	157.5 ± 4.7
*p*NPL	79.1 ± 9.1	Cu^2+^	135.1 ± 1.4	Cu^2+^	76.4 ± 5.8	1.5M	119.6 ± 5.4
*p*NPD	56.6± 3.3	Cr^2+^	147.4 ± 5.4	Cr^2+^	65.4 ± 5.5	2 M	157.5 ± 4.7
*p*NPC	32.1 ± 0.6	Mg^2+^	59.5 ± 0.1	Mg^2+^	17.1 ± 0.7	2.5M	164.7 ± 3.1
*p*NPB	28.4± 3.7	Mn^2+^	163.5 ± 5.0	Mn^2+^	42.4 ± 5.3	3M	170.3 ± 2.6
*p*NPA	23.5 ± 4.3	K^+^	106.4 ± 5.6	K^+^	30.5 ± 6.5	3.5M	164.6 ± 5.3
**Temperature**		Ca^+^	172.4 ± 0.5	Ca^+^	123.2 ± 0.3	4M	217.1 ± 0.8
4^o^C	57.6 ± 5.1	Ni^2+^	98.1 ± 1.8	Ni^2+^	55.6 ± 5.7	4.5 M	226.2 ± 0.1
10^o^C	57.7 ± 2.1	Fe^3+^	74.1 ± 1.2	Fe^3+^	72.1 ± 0.6	5M	234.3 ± 2.1
20^o^C	63.5 ± 9.9	Pb^2+^	76.2 ± 1.9	Pb^2+^	58.1 ± 0.6	**Detergents & others**	
30^o^C	72.4 ± 3.6	Cd^2+^	67.2 ± 1.3	Cd^2+^	53.7 ± 4.6	EDTA (0.1%)	86.6 ± 4.9
40^o^C	100.0 ± 1.2	Hg^2+^	62.3 ± 2.5	Hg^2+^	49.3 ± 1.5	Triton X (0.1%)	69.2 ± 1.8
50^o^C	92.7 ± 2.9	Ba^2+^	29.1 ± 0.1	Ba^2+^	09.7 ± 5.0	Tween 80 (0.1%)	121.5 ± 4.7
60^o^C	89.3 ± 2.7	Zn^2+^	88.1 ± 1.2	Zn^2+^	90.2 ± 3.4	SDS (0.1%)	32.2 ± 1.9
**pH**		Sn^2+^	34.1 ± 1.1	Sn^2+^	56.3 ± 5.2	CTAB (0.1%)	216.4 ± 3.4
pH3	62.3 ± 5.1	Li^+^	92.1 ± 1.1	Li^+^	78.0 ± 0.6	Gum arabic (0.1%)	75.6 ± 5.2
pH4	62.0 ± 0.6	Rb^+^	36.2 ± 1.7	Rb^+^	23.4 ± 1.0	DTT (0.1mM)	32.1 ± 0.6
pH5	54.7 ± 4.6	Cs^+^	78.1 ± 0.6	Cs^+^	36.0 ± 0.2	PMSF (0.1mM)	28.3 ± 2.4
pH6	68.3 ± 5.7	Al^3+^	53.1 ± 1.2	Al^3+^	32.3 ± 5.7	EDTA (1%)	33.2 ± 1.9
pH7	100.0 ±1.5	Co^2+^	56.5 ± 4.0	Co^2+^	28.3 ± 5.7	Triton X (1%)	16.4 ± 3.6
pH8	57.1 ± 1.1	CH_3_COO^-^	74.4 ± 5.6	CH_3_COO^-^	57.1 ± 1.4	Tween 80 (1%)	48.3 ± 2.5
pH9	73.3 ± 5.7	NO_3_^-^	82.1 ± 0.7	NO_3_^-^	64.1 ± 0.8	SDS (1%)	21.2 ± 1.8
pH10	84.3 ± 5.7	Cl^-^	73.1 ± 0.2	Cl^-^	89.2 ± 2.3	CTAB (1%)	102.2 ± 1.9
pH11	52.7 ± 1.5	SO_3_^2-^	83.5 ± 1.2	SO_3_^2-^	72.8 ± 5.4	Gum arabic (1%)	18.1 ± 1.2
pH12	50.6 ± 5.3	PO_4_^3-^	78.1 ± 4.3	PO_4_^3-^	85.3 ± 9.2	DTT (1mM)	11.4 ± 3.8
						PMSF (1mM)	4.6 ± 5.0

Mean relative activity values are shown with *p*- nitrophenyl palmitate as substrate. Values represent the mean of at least 3 separate determinations.

#### Effect of pH and temperature on Lpc53E1 activity

Lpc53E1 activity was measured within the pH range of 3–12, to determine the optimal pH for lipase activity. An optimum activity of 1,900 U/mg protein had previously been determined at pH 7 (100% relative activity), (Table 1). Lpc53E1 displayed activity over a wide range of pH values ranging from 62% relative activity at pH3 (1,200U/mg) to 84% of relative activity at pH 10 (1,600 U/mg). Lpc53E1 also displayed activity over a broad temperature range from 4 to 60°C (Table 1). While displaying a temperature optimum of 40°C (100% relative activity), Lpc53E1 retained nearly 92% and 89% relative activity at 50°C and 60°C respectively. Good levels of activity were also observed at lower temperatures with almost 58% relative activity at 4°C.

#### Effect of NaCl and other metal ions on Lpc53E1 activity

The recombinant lipase Lpc53E1 displayed increased levels of activity upon exposure to increased concentrations of NaCl, from 1 M (157% relative activity) up to a level of 5 M (Table 1); where activity was 234% relative to the control without NaCl addition. Various metal ions had differing effects on enzyme activity, with most of the anions tested having a negative effect on relative enzyme activity. The addition of 1 mM and 10 mM of Sn^2+^ and Ba^2+^, 10 mM of Rb^+^ and Mg^2+^ having the most marked effects on lipase activity. In contrast however exposure to 1 mM concentrations of Ag ^+^, Ca^2+^, Mn^2+^, Cu^2+^ and Cr^2+^ resulted in increased levels of relative enzyme activity, an effect that was however reversed when the concentrations of these metal ions were increased to 10 mM (Table 1). The addition of Ca^2+^ resulted in the biggest increase in activity to 72% more than the control (with no metal addition), an effect which was still evident at 10 mM Ca^2+^ where an increase of 23% was observed relative to the control.

#### Effects of detergent and other reagents on Lpc53E1 activity

The addition of various detergents and other reagents had varying effects on lipase activity. The addition of Tween 80 (0.1%) and cetyltrimethylammonium bromide (CTAB) (0.1%) resulted in increases in relative enzyme activity of 1.2 and 2 fold respectively compared to the control activity which had no detergent added. On the other hand increasing the Tween 80 concentration to 1% reduced enzyme activity by 83.5% while the addition of Triton X (0.1%) and SDS (0.1%) resulted in 30% and 67% reductions in relative enzyme activity respectively. The most marked reduction in relative activity was observed upon addition of inhibitors such as dithiothreitol (DTT) and p-methylphenyl sulfonylfluoride (PMSF) which reduced activity by 88% and 95% respectively (Table 1). Gum arabic at 0.1% resulted in the retention of nearly 76% activity but at 1% enzymatic activity was significantly reduced. Both concentrations (0.1 and 1%) of gelatin resulted in the retention of nearly 50% of the enzymatic activity.

#### Determination of optimum Lpc53E1 assay condition by RSM

The concentrations of four variables (A: *p*NPP (mM), B: Ca^2+^(mM), C: Reaction time and D: NaCl) for optimum lipase assay conditions were determined by analyzing the responses in detail for all possible combinations with two factors being constant at a time using the point prediction feature of the design expert software. Thirty experiments were performed and the relative Lpc53E1 activity obtained in both predicted and actual values were determined (Additional file 1). The predicted responses of the relative lipase activity were found to be very close to the experimental values, which indicated that the generated model was an adequate prediction of the optimum Lpc53E1 assay conditions. The optimum pH 7 and temperature 40°C was set as constant, and the remaining selected four variables variables (A: *p*NPP (mM): -1(0.5), 0 (1.0), +1(1.5), B: Ca^+^(mM): -1(0), 0 (5), +1(10) C: Reaction time (Min): -1 (5), 0 (15), +1 (25) and D: NaCl (M) : -1 (4), 0(5), +1(6)). These experiments were performed and the responses were analyzed, following cubic regression polynomial equations were obtained:

$$\begin{array}{ll} \text{Relative activity}\left(\text{Y}\right)= & +100.00+1.4*\text{ A}+3.0*\text{ B}\\ & +1.57\;*\text{ C}+2.16\;*\text{ D}-4.57\;\\ & *\text{ A }*\text{ B}+4.79*\text{ A }*\text{B}+4.79\\ & *\text{ A }*+2.34*\text{ A }*\text{ D}+1.59*\text{ B }\\ & *\text{ C}+3.18*\text{ B }*\text{ D}+8.00*\text{ C }\\ & *\text{ D}-10.66*{\text{ A}}^{2}-10.2\;*\text{ B}\\ & -4.58-8.36*{\text{ D}}^{2} \end{array}$$

The significance of each coefficient was determined by *F*-values and *P*-values. The *P*-values suggest that, among the test variables used in this study AB (*p*NPP * Ca^+^), AC (*p*NPP * reaction time), CD (reaction time * NaCl), A^2^ (*p*NPP * *p*NPP) B^2^ (Ca^+^ * Ca^+^), C^2^ (reaction time * reaction time) and D^2^ (NaCl* NaCl) are significant model terms with *P*-values of < 0.0001 and the other terms are significant with *P* > 0.05 (Additional file 1). Based on the *F*- value and *P*- value the model is predictive with the regression coefficient (0.9818), which is close to unity indicating a good reproducibility with the experimental data. The predicted responses of the relative lipase activity were very close to the experimental values, which indicated that the generated model was an adequate prediction of the optimum Lpc53E1 assay conditions.

**Figure 4:**
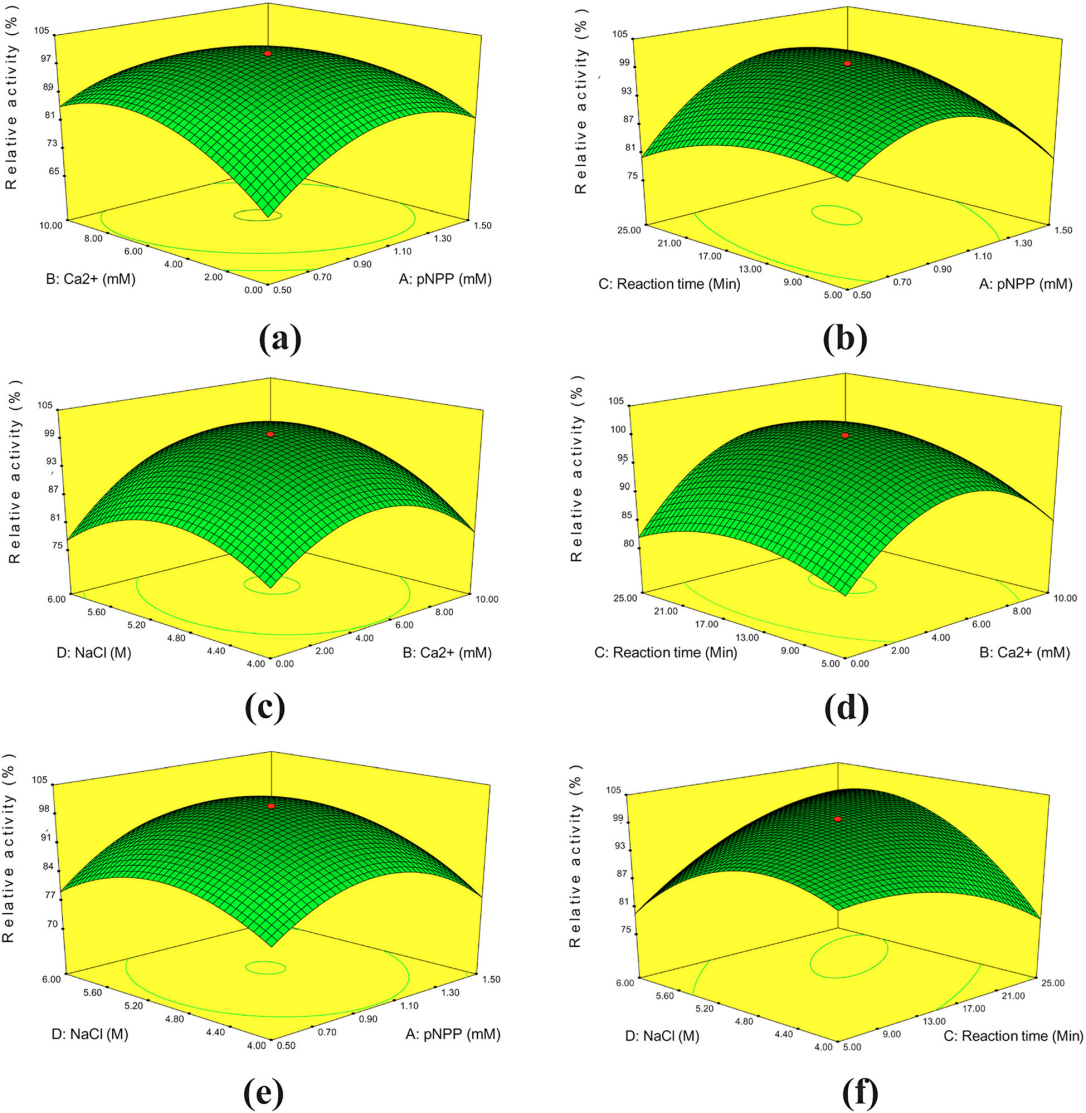
**3-D contour plots of relative enzyme activity with four variables (A: pNPP (mM), B: Ca**^**2+**^**(mM), C: Reaction time and D: NaCl).a)**. Ca^2+^(mM) and *p*NPP (mM), **b)**. Reaction time (min) and *p*NPP (mM), **c)**. NaCl (M) and Ca^2+^(mM), **d)**. Reaction time (min) and Ca^2+^ (mM), **e)**. NaCl and *p*NPP (mM) and **f)**. NaCl (M) and Reaction time (min). The optimum Lpc53E1 activity of 2700U/mg at 1 mM pNPP, 5 mM Ca^2+^, 5 M NaCl at reaction time of 15 min, at pH 7 and temperature of 40°C predicted from the surface graphs.

Three-D contour plots were also generated delineating the predicted responses over a range in the design surface (Figure 4a- f). In 3D contours, the responses were studied with two factors at a time while the remaining variables remaining constant. At the applied point prediction aspect, the maximum lipase assay conditions were obtained when pNPP and Ca^2+^ and NaCl were at concentrations of 1 mM, 5 mM and 5 M respectively, at a reaction time of 15 min, with the pH and temperature being constant at 7 and 40°C respectively. Under these conditions the lipase activity was determined as 2,700 U/mg.

#### Kinetics of Lpc53E1 lipase activity

The kinetic constants K_M_ and *Vmax* were determined for the purified recombinant lipase by employing a Lineweaver – Burk plot with *p*NPP as substrate and at pH7 and 40°C K_M_ and *Vmax* values of 1.093 mM^-1^ and 50 μmol/min, respectively were obtained (Additional file 1). The high affinity of the recombinant enzyme for *p*NPP is reflected in the relatively low K_M_ value.

#### Stability profile of Lpc53E1

The thermal stability of the recombinant Lpc53E1 protein was assessed following incubation for 1 h at a variety of temperatures ranging from 4°C to 90°C (Table 2). The enzyme is quite stable at higher temperatures (60–80°C) retaining up to 85% of its original activity at 80°C and almost 57% activity at 90°C. It shows good stability at temperatures from 4°C to 60°C. The pH stability of the enzyme was assessed following incubation for 24 h at 4°C at pH ranging from 3–12 in different buffer systems. The recombinant Lpc53E1 lipase was stable over a range of pH values retaining 54% activity at the lowest pH 3, while retaining around 79% and 74% residual activity at pH 9 and pH 10 respectively (Table 2).

**Table 2:** Stability profile of Lpc53E1 with pH, temperature, solvents and NaCl

***Variables***	***Residual activity (%) (SD(yEr-))***	***Variables***	***Residual activity (%) (SD(yEr-))***	***Variables***	***Residual activity (%) (SD(yEr-))***
**pH**		**Solvents (10%)**		**NaCl**	
Standard	100.0 ± 0.2	Control	100.0 ± 0.6	Standard	100.0 ± 0.6
pH3	54.6 ± 2.7	Methanol	126.1 ± 1.8	0M	100.0 ± 0.6
pH4	59.1 ± 1.8	Acetone	90.1 ±1.3	1M	98.4 ± 1.2
pH5	55.2 ± 3.9	n-hexane	111.4 ± 5.8	2 M	92.3± 4.2
pH6	48.1 ± 0.1	2-Propanol	101.0 ± 0.7	3 M	93.2 ± 2.6
pH7	100 ± 0.3	Butanol	122.3 ± 4.6	4M	92.3 ± 4.1
pH8	82.3 ± 0.1	Ethanol	119.0 ± 0.3	5M	96.3 ± 3.7
pH9	79.2 ± 2.4	DMSO	126.1 ± 1.8		
pH10	74.0 ± 0.5	Glycerol	270.3 ± 3.9		
pH11	48.1 ± 1.0	Acetonitrile	155.1 ± 0.8		
pH12	37.1 ± 0.2				
**Thermostability**		**Solvents (20%)**			
Standard	100.0 ± 0.4	Control	100.0 ± 0.5		
10^o^C	98.9 ± 3.6	Methanol	220.5 ± 6.7		
20^o^C	96.3 ± 2.3	Acetone	157.4 ± 5.8		
30^o^C	94.2 ± 5.3	n-hexane	195.4 ± 4.9		
40^o^C	99.6 ± 0.6	2-Propanol	176.4 ± 5.4		
50^o^C	94.5 ± 3.0	Butanol	473.2 ± 3.4		
60^o^C	90.1 ± 6.8	Ethanol	208.2 ±2.3		
70^o^C	88.6 ± 5.1	DMSO	220.5 ± 6.7		
80^o^C	85.1 ± 3.0	Glycerol	214.3 ± 4.5		
90^o^C	57.6 ± 5.2	Acetonitrile	271.2 ± 3.2		

The mean relative activity and standard deviation of values are shown. All experiments were conducted spectrophotometrically with *p*- nitrophenyl palmitate as a substrate. Reference standard was set with 4°C, pH 7.0 and 5 M NaCl to determine residual activity under standard assay conditions for temperature, pH and NaCl respectively. Without solvent was included as reference standard for calculating residual activity of organic solvents.

The solvent stability of the recombinant lipase was assessed following exposure to either 10% or 20% of a variety of solvents for a 24 h period at 4°C. The stability of the lipase was largely unaffected by solvent addition, except in the case of 10% acetone where around 10% activity was lost. In all other cases the stability of Lpc53E1 actually increased upon exposure to solvents relative to the control. This was particularly evident upon exposure to 10% glycerol and 20% butanol, where large increases in residual activity levels were observed (Table 2).

#### Stability in higher salt concentration

It was noted that the optimum Lpc53E1 activity was attained at 5 M NaCl, compared to control without salt. The increased level of enzyme activity at higher NaCl levels (Table 1) was mirrored in the halotolerance observed in Lpc53E1 upon exposure to various NaCl concentrations for 24 h at 4°C. Nearly 99% residual activity was observed following incubation with 5 M NaCl and small decreases in residual activity were observed at lower salt concentrations, with 1 M NaCl having the most marked effect on activity, but even then around 92% residual activity was observed (Table 2). This high salt stability profile could make this lipase particularly useful for many industrial applications, particularly those related to the production of marine products.

## Discussion

A novel extremely halotolerant lipase Lpc53E1 has been identified following the functional screening of a *H. simulans* metagenomic library and the recombinant enzyme has subsequently been biochemically characterized. Functional screening of soil and sediment metagenomic libraries have resulted in the discovery of several new lipases, including one halotolerant lipase from fat contaminated soil collected from a wastewater treatment plant, which displayed salt tolerant capability up to 3.7 M NaCl and an optimum lipolytic activity at 1.7 M NaCl [22] . New families of lipolytic enzymes, most with a specificity for short chain fatty acids, have also been isolated and identified from marine metagenomes, including *LipG*, from a Korean tidal flat sediments metagenomic library [15]; *EstA*, from a South China Sea surface water metagenomic library [23]; *LipEH166*, from an intertidal flat metagenomic library [24], *EstF*, from a South China Sea marine sediment metagenomic library [25] and salt-tolerant esterases from tidal flat sediment in South Korea [26].

The biochemical characterization of the recombinant protein Lpc53E1 protein revealed that it was active with longer chain fatty acyl esters as substrates such as *p*-nitrophenyl palmitate (C16), *p*-nitrophenyl myristate (C14) and p-nitrophenyl laurate (C12) (Table 1). This preference for longer chain length fatty acid esters indicates that Lpc53E1 is a lipase family member as opposed to an esterase. With *p*-NPP as substrate the enzyme was active over the pH range 6 to 11 and at temperatures from 40 to 80°C. Lpc53E1 displayed maximum activity at pH 7 and at 40°C. The stability of Lpc53E1 over a wide range of pH and temperature (Table 2) indicate that the enzyme is both alkaliphilic and thermostable.

Lpc53E1 activity was enhanced in the presence of various metal ions, but in particular in the presence of Ca^2+^. Metal ions are known to enhance enzyme activity by increasing the structural stability of the protein by binding to negatively charged amino acid residues [27]. The observed increase in Lpc53E1 activity in the presence of Ca^2+^ ions is similar to the increases observed in the novel lipases isolated from the tidal flat sediment metagenomic library [15] and the lipase from the fat contaminated soil metagenomic library [22]. Ca^2+^ ions are believed to act as ligands between amino acid residues in the enzyme active site [28] and the electrostatic interactions between calcium and fatty acids produced by the hydrolysis of the substrate leads to the “clearing” of the active site thereby allowing another substrate molecule to access the site [29].

Incubation of Lpc53E1 in the presence of organic solvents (Table 2) increased enzyme activity. This is a well established phenomenon with lipases with for example lipases from the aforementioned fat contaminated soil metagenomic library, displaying similar characteristics [22]. In the well studied *Candida rugosa* system, lipase activation by organic solvents is mediated by the solvent keeping the lid of the enzyme in an open confirmation, thereby facilitating access by the substrate to the enzyme active site [30]. Thus the increased Lpc53E1 activity in the presence of organic solvents suggests that the enzyme may possess a lid that is converted into an open conformation in the presence of various solvents.

We employed RSM analysis to determine the optimum conditions for Lpc53E1 activity. This approach considers both the effect of primary factors and their mutual interactions in a multivariate system [31]. RSM analysis can predict the overall enzyme behavior with limited experimental points, without any knowledge of enzymatic mechanisms. The RSM- CCD analysis with four factors-3- level fractional design employed in this study was efficient in reducing both the experiments required and the time necessary to investigate the optimal conditions for Lpc53E1 activity.

Lpc53E1 is a novel family VIII lipase, containing the highly conserved β-lactamase active site motif SXTK. The inhibition of the enzyme by PMSF confirms the involvement of an active site serine in the reaction mechanism. The protein is related to other hydrolytic enzymes of known function together with a number of lipolytic enzymes also identified from metagenomic sources. However phylogenetic analysis indicates that Lpc53E1 is a member of a separate cluster within the family VIII lipases and is most closely related to a group of uncharacterized proteins identified as putative β-lactamases. Lpc53E1 lacks β-lactamase activity and, on the basis of this phylogenetic analysis, it seems likely that the other proteins within the Lpc53E1 cluster may also possess lipolytic activity.

## Conclusion

We have cloned a novel lipase following a functional screen of a marine sponge metagenomic library. Sequence analysis indicated that Lpc53E1 is a member of the group VIII family of lipases. While the substrate specificity of other metagenome-derived family VIII lipases has predominantly been for short chain fatty acyl esters, the low K_M_ value for long chain fatty acyl esters, coupled with broad activity, thermal and solvent stability, and very high halotolerance expands the potential utility of this enzyme in various industrial applications. This study also highlights the advantages of marine metagenomic libraries for cloning novel genes through functional based approaches. Such approaches should prove useful in the future identification of other biocatalysts and biomolecules with potential utility in various biotechnological applications.

## Materials and methods

### Metagenomic library construction and screening of lipase clone

The total community DNA was extracted from microbial population associated with the marine sponge *Haliclona simulans* collected by SCUBA in Kilkieran Bay, off the west coast of Ireland. Following pulse-field-gel-electrophoresis, a size fractionated ~40 kb insert was cloned into the fosmid Copy Control pCC1FOS™ vector to construct a metagenomic library. Following *in vitro* packaging into lambda phages and infection of *E.coli* TransforMax™ EPI300™, clones were transferred to 384 well plates using the QPIX2-XT robot. The library was replicated onto agar Q-Tray plates containing 1% tributyrin and positive clones identified by the presence of clear halos. The positive clones showing highest lipase activity on tributyrin plates were selected for further work.

### Pyrosequencing and CAMERA analysis

The pCC1Fos53E1 was sequenced by Roche 454 pyrosequencing with a pool of other metagenomic fosmid clones. Sequencing and assembly were carried out by the University of Liverpool, Centre for Genomic Research. Contigs from the assembly were interrogated for the presence genes encoding potential lipolytic activities using the RAMMCAP pipeline [32] hosted by CAMERA [33]. Contigs and potential ORFs were further analysed by BLAST and ORF predictions were refined.

### Lipase sequence analysis and phylogenetic tree construction

The solubility and membrane protein probability of the protein was predicted by performing SOSUI system analysis [34]. Multiple sequence alignment was carried out using the Clustal W algorithm in combination with MEGA version 5.0 Software. The protparam tool was used to calculate the theoretical parameters of the protein [35]. Evolutionary relatedness of the protein was analyzed using MEGA version 5.0 and the evolutionary history was inferred using the Neighbor-Joining method [36] with the bootstrap test (1000 replicates) [37].

### Cloning of Lpc53E1 gene

Primers were designed to amplify the full length coding sequence of *lpc*53E1 with restriction sites incorporated to allow in-frame cloning into the pBAD/mycHIS-A vector. Primer 4.5f (5′-TATATATC*ATGA*AATCGAGGCTGAACGACATCC-3′) incorporates a *Pag*I restriction site (underlined) at the predicted start codon (italics). Reverse primer 4.5r (5′-ATATATAAGCTTTGCCAGTGATTGATACACCGCTCG-3′) incorporates a *Hind*III restriction site (underlined) replacing the predicted stop codon. The ORF of the putative lipase gene was then PCR amplified and purified. The PCR conditions were as follows: 94°C for 3 min followed by 35 cycles at 94°C for 45 s, 65°C for 45 s, 72°C for 60s and a final extension at 72°C for 10 min. The PCR -amplified DNA fragments were confirmed by electrophoresis in 2% agarose and visualized following ethidium bromide staining. The amplified gene was digested with *Pag*I and *Hin*dIII (Fermentas) and ligated to the pBAD/*Myc*-HisA expression vector (Invitrogen) that had been digested with *Nco*I and *Hin*dIII. Recombinants were transformed into *E. coli* TOP10 cells and clones were assayed on tributyrin plates for lipase activity. The correct DNA sequence of the *lpc*53E1 gene was confirmed.

### Recombinant expression and lipase protein purification

The transformed cells carrying the pBAD/*Myc*-HisA vector containing the *lpc*53E1 gene were grown in 250 ml of LB medium containing 50 μg/ml concentration of ampicillin at 37°C for overnight incubation in rotary shaker (250 rpm) to reach the OD (at 600 nm) of 0.6. The culture was then induced with 10 μg/ml concentration of L-arabinose for expression of the Lpc53E1 protein. After 4 h incubation, the cells were centrifuged (5000 × g for 10 min) at 4°C. The cell pellet was resuspended in 10 ml guanidinium lysis buffer (6 M guanidine hydrochloride, 20 mM sodium phosphate, pH 7.8, 500 mM NaCl pH 7.8) and cells were incubated for 5–10 min at room temperature to ensure thorough cell lysis. Cells were then disrupted by sonication in ice with three 5- second pulses at high intensity. The cell lysate was centrifuged at 3,000 × g for 15 min to pellet the cellular debris and collect the supernatant containing the His- tagged Lpc53E1 protein. The supernatant was then loaded on a ProBond™ Column with Ni^2+^ resin (Invitrogen). The column was washed twice with denaturing binding buffer (8 M urea, 20 mM sodium phosphate pH 7.8 and 500 mM NaCl) and twice with denaturing wash buffer (8 M urea, 20 mM sodium phosphate, pH 6.0 and 500 mM NaCl). Before eluting the His- tagged lipase with the elution buffer (250 mM NaH_2_PO4, pH 8.0, 2.5 M NaCl, 250 mM imidazole) the column was washed with native wash buffer (250 mM NaH_2_PO4, pH 8.0, 2.5 M NaCl, 20 mM imidazole). The collected fractions were pooled and concentrated by using Centricon concentrators, and further dialyzed against double distilled water with lipase activity being confirmed on tributyrin agar plates prior to SDS- PAGE analysis.

### Characterization of Lpc53E1 activity with *p*-nitrophenyl palmitate as substrate

The activity of purified Lpc53E1 was measured spectrophotometrically with the long chain fatty acyl ester *p*- nitrophenyl palmitate (C_16_) as substrate at λmax 405 nm [38]. Reactions were performed in triplicate with continuous monitoring of *p*-nitrophenol liberation over a period of 25 min at 40°C in a microplate spectrophotometer. The *p*-nitrophenyl acyl substrate analogs were hydrolyzed to yield the fatty acid and *p*-nitrophenol, which is a chromophore absorbing light at λmax 400 to 410 nm in slightly alkaline media (pKa = 7.2) with a Molar extinction coefficient of 1.78 × 10^4^ M^-1^ × cm^-1^ at 405 nm. All reactions were performed in a microtitre plate at a total volume of 250 μl; with a final concentration of 1 mM *p*NPP, 5 nM purified lipase, 50 mM Tris- HCl pH 8.2, 1 mM CaCl_2_, 0.3% (v/v) Triton X-100, 4% (v/v) isopropanol and 1% (v/v) acetonitrile containing 230 μl of reaction mixture and 20 μl of purified lipase [23]. One unit of lipase activity was defined as the amount of enzyme releasing 1 μmol of *p*-nitrophenol per min at 40°C under standard reaction conditions.

### Effect of various physiochemical parameters on lipase activity

#### Substrate specificity of Lpc53E1

The substrate specificity of the lipase was assessed by employing *p*- nitrophenyl esters with acyl chains of different lengths in the range of C_2_- C_16_ such as *p*-nitrophenyl acetate, *p*-nitrophenyl butyrate, *p*-nitrophenyl caprylate, *p*-nitrophenyl decanoate, *p*-nitrophenyl laurate, *p*-nitrophenyl myristate and *p*-nitrophenyl palmitate. For each assay all substrates were prepared from a 20 mM stock of appropriate substrate in 4:1 ratio of isopropanol and acetonitrile. The substrate solution was obtained with an assay buffer of 50 mM Tris–HCl pH 7.5, 1 mM CaCl_2_, 0.3% Triton X under agitation at 60°C until the formation of clear transparent solution [22]. For each assay 230 μl of the appropriate substrate and 20 μl of lipase solution prepared in 50 mM of phosphate buffer pH 8 were pipetted into a 96 well microtitre plate. After the reaction time of 25 min at 40°C, the λmax at 405 nm was determined in a micro plate spectrophotometer.

#### Effects of temperature on Lpc53E1 activity

The effect of temperature on Lpc53E1 activity was determined by incubating the lipase with the substrate *p*-nitrophenyl palmitate in the temperature range of 4–60°C. The reaction mixture was prepared with 10 mM *p*NPP, 50 mM Tris HCl pH 8.2, 1 mM CaCl_2_, 0.3% (v/v) Triton X-100, 4% (v/v) isopropanol and 1% (v/v) acetonitrile in the final assay substrate solution of 200 μl and the 20 μl of 5 nM enzyme solution was prepared in phosphate buffer, pH of 8.2. All reactions were carried out in the 96 well PCR plate with at 25 min reaction time over the aforementioned temperature range. The reactions were stopped by adding 30 μl chilled acetone: ethanol in the ratio of 1:1, which was kept at −20°C and λmax of 405 nm, was determined.

#### Effects of pH on Lpc53E1 activity

The optimal pH of lipase activity was investigated with *p*- nitrophenyl palmitate as substrate in different pH buffer solutions at 40°C. A series of buffer systems representing different pH ranges were prepared including 50 mM sodium acetate buffer (pH 3–6), 50 mM of phosphate buffer (pH 7), and 50 mM glycine-NaOH buffer (pH 8–12). All reactions were prepared in 96 well PCR microtitre plates as previously described with 5 nM enzyme solution in appropriate buffer. The reaction mixture and enzyme solution were incubated at various pH as specified above for 25 min, and the λmax at 405 nm determined using a micro plate spectrophotometer.

#### Effects of metals on Lpc53E1 activity

Lpc53E1 activity was determined at NaCl concentrations ranging from 0.5 to 5 M. All the assays were carried out in 96 well microtitre plate as previously described with 20 nM of enzyme solution under standard assay condition at 40°C for 25 min. The effect of different metals ions on lipase activity was assessed by addition of 25 μl of either 1 mM and 10 mM concentrations of various metals (Ag^+^, Cu^2+^, Cr^2+^, Mg^2+^, Mn^2+^, K^+^, Ca^2+^, Ni^2+^, Fe^3+^, Pb^2+^, Cd^2+^, Hg^2+^, Ba^2+^, Zn^2+^, Sn^2+^, Li^+^, Rb^+^, Cs^+^, Al^3+^, Co^2+^, CH_3_COO^-^, NO_3_^-^, Cl^-^, SO_3_^2-^ and PO_4_^3-^ to 25 μl of 50 nM enzyme solution prepared with 200 mM of Tris – HCl buffer pH 8.0. All metal solutions were prepared in the form of chloride salts except Ag^+^ (nitrate salt) and Cu^2+^ (sulphate salt). The reaction mixtures were prepared with 200 μl of substrate solution as described earlier with 50 μl of enzyme- metal solutions. 250 μl of reaction mixtures in 96 well microtitre plates were incubated at 40°C for 25 min and lipase activity was monitored at 405 nm using a micro plate spectrophotometer. The relative activities were calculated against a control to which no metal solution had been added.

#### Effects of inhibitors and detergents on Lpc53E1 activity

The effect of lipase activity was also tested with a number of detergents and inhibitors Triton X, Tween 80, SDS,CTAB and EDTA at concentrations of 0.1% and 1% and dithiothreitol (DTT), p- methylphenyl sulfonylfluoride (PMSF) at concentrations of 0.1 mM and 1 mM. The effect of the addition of emulsion stabilizer gum arabic and solidifier gelatin on lipase activity was also determined. All the assays were carried out in 96 well microtitre plate as previously described with 20 nM of enzyme solution under standard assay condition at 40°C for 25 min.

#### Effects of NaCl concentration on Lpc53E1 activity

Lpc53E1 activity was determined at NaCl concentrations ranging from 0.5 to 5 M. All the assays were carried out in 96 well microtitre plate as previously described with 20 nM of enzyme solution under standard assay condition at 40°C for 30 min.

#### Statistical analysis by RSM and origin 8 software packages

The response surface method (RSM) was employed to determine the optimal reaction conditions for Lpc53E1. This was achieved by analyzing the responses in detail for all possible combinations of the four variables using the point prediction feature of the design expert software version 8.0.6.1 (state ease USA). While the optimum pH 7 and temperature 40°C was set as constant, four selected variables were applied on CCD (Circular) analysis to predict optimum assay conditions; namely pNPP, Ca^2+^ and NaCl concentrations together with various reaction times. The statistical analyses of linear, quadratic and cross-product effects were evaluated by the second order polynomial response surface equation using CCD to reach optimum lipase assay conditions with four variables.

All statistical analysis (SD) were performed in triplicate values obtained from each experiment by origin 8 software packages.

### Stability studies of Lpc53E1 activity

#### pH stability of Lpc53E1

The pH stability of the lipase enzyme was evaluated by incubating 50 nM purified Lpc53E1 for 24 h at 4°C in different buffer systems of 50 mM sodium acetate (pH 3–6), 50 mM of phosphate buffer (pH 7), and 50 mM of glycine -NaOH buffer (pH 8–12). After 24 h of incubation, the residual activity was calculated under standard assay conditions with pH 7 as a reference standard.

#### Thermostability of Lpc53E1

The thermostability of Lpc53E1 was determined by incubating 25 μl of 50 nM enzyme with 25 μl of 50 mM phosphate buffer pH 8 at the temperature range of 4–90°C for 1 h. All reactions were performed in 96 well PCR plates with the addition of mineral oil to prevent evaporation [25]. Incubation temperatures were achieved by employing the gradient mode on the PCR thermal cycler. Before residual activity measurement, the reaction tubes were chilled on ice.

#### Solvent stability of Lpc53E1

The organic solvent stability of Lpc53E1 was assessed by measuring the residual activity after incubating the enzyme solution with 10% and 20% concentrations of different solvents (methanol, ethanol, acetone, n- hexane, 2- propanol, butanol, DMSO, glycerol and acetonitrile). All reaction mixtures were incubated at 4°C for 24 h with 50 nM of enzyme and appropriate concentrations of different solvents. The control without solvent added was taken to calculate the residual activity.

#### Stability at high concentration of salt

The stability of Lpc53E1 at different salt concentrations was determined by measuring the residual activity after incubating the enzyme solution (50 nM enzyme, 50 mM Tris–HCl pH 7.5 and 1 mM CaCl_2_) with different molar concentration of NaCl in the range of 1–5 M for 24 h at 4°C. The residual activity was calculated as previously described.

## Competing interests

The authors declare that they do not have any competing interests.

## Authors’ contributions

JS, JK, DPHL, and SG performed the experiments, JS, JK, and ADWD designed the experimental approach and wrote the manuscript. All authors read and approved the final manuscript.

## Supplementary Material

Additional file 1 Supplementary files. Click here for file

## References

[B1] Siddiqui KS Cavicchioli R Cold-adapted enzymes Annu Rev Biochem 2006 75 403 433 10.1146/annurev.biochem.75.103004.142723 16756497

[B2] Trincone A Marine Biocatalysts- Enzymatic Features and Applications Mar Drugs 2011 9 478 499 10.3390/md9040478 21731544 PMC3124967

[B3] Webster NS Taylor: Marine sponges and their microbial symbionts: love and other relationships Environ Microbiol 2011 [Epub ahead of print] 10.1111/j.1462-2920.2011.02460 21443739

[B4] Wilkinson CR Microbial association in sponges II: numerical analysis of sponge and water bacterial populations Mar Biol 1978 49 169 176 10.1007/BF00387116

[B5] Kennedy J Leary ND Kiran GS Morrissey JP Gara F Selvin J Dobson ADW Functional metagenomic strategies for the discovery of novel enzymes and biosurfactants with biotechnological applications from marine ecosystems J Appl Microbiol 2011 111 787 799 10.1111/j.1365-2672.2011.05106.x 21777355

[B6] Amann RI Ludwig W Schleifer KH Phylogenetic identification and in situ detection of individual microbial cells without cultivation Microbiol Rev 1995 59 1 143 169 7535888 10.1128/mr.59.1.143-169.1995 PMC239358

[B7] Kennedy J Marchesi JR Dobson ADW Marine metagenomics: strategies for the discovery of novel enzymes with biotechnological applications from marine environments Microb Cell Fact 2008 7 2 10.1186/1475-2859-7-2 18717988 PMC2538500

[B8] Simon C Daniel R Metagenomic analyses: past and future needs Appl Environ Microbiol 2011 77 1153 1161 10.1128/AEM.02345-10 21169428 PMC3067235

[B9] Ferrer M Beloqui A Timmis KN Golyshin PN Metagenomics for mining new genetic resources of microbial communities J Mol Microbiol Biotechnol 2009 16 109 123 10.1159/000142898 18957866

[B10] Steele HL Jaeger KE Daniel R Streit W Advances in recovery of novel biocatalysts from metagenomes J Mol Microbiol Biotechnol 2009 16 25 37 10.1159/000142892 18957860

[B11] Venter JC Remington K Heidelberg JF Halpern AL Rusch D Eisen JA Wu D Paulsen I Nelson KE Nelson W Environmental genome shotgun sequencing of the Sargasso Sea Science 2004 304 66 74 10.1126/science.1093857 15001713

[B12] Jaeger KE Eggert T Lipases for biotechnology Curr Opin Biotechnol 2002 13 390 397 10.1016/S0958-1669(02)00341-5 12323363

[B13] Arpigny JL Jaeger KE Bacterial lipolytic enzymes: classification and properties, Biochem J 1999 343 177 183 PMC1220539 10493927

[B14] Henne A Schmitz RA Bomeke M Gottschalk G Screening of environmental DNA libraries for the presence of genes conferring lipolytic activity on Escherichia coli Appl Environ Microbiol 2000 66 3113 3116 10.1128/AEM.66.7.3113-3116.2000 10877816 PMC92121

[B15] Lee MH Lee CH Oh TK Song JK Yoon JH Isolation and characterization of a novel lipase from a metagenomic library of tidal flat sediments: evidence for a new family of bacteria lipases Appl Environ Microb 2006 72 7406 7409 10.1128/AEM.01157-06 PMC1636159 16950897

[B16] Karpushova A Brummer F Barth S Lange S Schmid RD Cloning, recombinant expression and biochemical characterization of novel esterases from *Bacillus* sp. associated with the marine sponge *Aplysina aerophoba* Appl Microl Biotechnol 2005 67 59 69 10.1007/s00253-004-1780-6 15614567

[B17] Okamura Y Kimura T Yokouchi H Meneses-Osori M Katoh M Matsung T Takeyama H Isolation and charcaterization of a GDSL esterase from the metagenome of a marine sponge-associated bacteria Mar Biotechnol 2010 12 395 402 10.1007/s10126-009-9226-x 19789923

[B18] Yung PY Burke C Lewis M Kjelleberg S Thomas T Novel antibacterial proteins from the microbial communities associated with the sponge *Cymbastela concentrica* and the green alga *Ulva australis* Appl Environ Microbiol 2011 77 1512 1515 10.1128/AEM.02038-10 21183639 PMC3067216

[B19] Hu Y Fu C Huang Y Yin Y Cheng G Lei F Lu N Li J Novel lipolytic genes from the microbial metagenomic library of the South China sea marine sediment FEMS Microbiol Ecol 2010 72 228 237 10.1111/j.1574-6941.2010.00851.x 20337707

[B20] Lejon DPH Kennedy J Dobson ADW de Bruijn F Identification of novel bioactive compounds from the metagenome of the marine sponge *Haliclona simulans* Handbook of Molecular Ecology II, Metagenomics in different habitats 2011 New Jersey: Wiley-Blackwell 553 562

[B21] Knox JR Moews PC Frere JM Molecular evolution of bacterial β-lactam resistance Chem Bio 1996 3 937 947 10.1016/S1074-5521(96)90182-9 8939710

[B22] Glogauer A Martini VP Faoro H Couto GH Santos MM Monteiro RA Mitchell DA Souza EM Pedrosa FO Krieger N Identification and characterization of a new true lipase isolated through metagenomic approach Microb Cell Fact 2011 10 54 10.1186/1475-2859-10-54 21762508 PMC3161859

[B23] Chu X He H Guo C Sun B Identification of two novel esterases from a marine metagenomic library derived from South China Sea Appl Microbiol Biot 2008 80 615 625 10.1007/s00253-008-1566-3 18600322

[B24] Kim EY Oh KH Lee MH Kang CH Oh TK Yoon JH Novel cold-adapted alkaline lipase from an intertidal flat metagenome and proposal for a new family of bacterial lipases Appl Environ Microb 2009 75 257 260 10.1128/AEM.01400-08 PMC2612223 18931297

[B25] Fu C Hu Y Xie F Guo H Ashforth EJ Polyak SW Zhu B Zhang L Molecular cloning and characterization of a new coldactive esterase from a deep-sea metagenomic library Appl Microbiol Biot 2011 90 961 970 10.1007/s00253-010-3079-0 21336688

[B26] Jeon JH Lee HS Kim JT Kim S-J Choi SH Kang SG Lee J-H Identification of a new subfamily of salt-tolerant esterases from a metagenomic library of tidal flat sediment Appl Microbiol Biotechnol 2011 PMID: 21720822 10.1007/s00253-011-3433-x 21720822

[B27] Colak A Pipik D Saglam N Guner S Canakci S Belduz AO Characterization of a thermoalkalophilic esterase from a novel thermophilic bacterium, *Anoxybacillus gonensis* G2 Bioresour Technol 2005 96 625 631 10.1016/j.biortech.2004.06.003 15501671

[B28] Salameh MA Wiegel J Purification and characterization of two highly thermophilic alkaline lipases from *Thermosyntropha lipolytica* Appl Environ Microbiol 2007 73 23 7725 7731 10.1128/AEM.01509-07 10.1128/AEM.01509-07 17933930 PMC2168070

[B29] Voget S Leggewie C Uesbeck A Raasch C Jaeger KE Streit WR Prospecting for novel biocatalysts in a soil metagenome Appl Environ Microbiol 2003 69 10 6235 6242 10.1128/AEM.69.10.6235-6242 14532085 PMC201203

[B30] Colton IJ Ahmed SN Kazlauskas RJ A 2-propanol treatment increases theenantioselectivity of *Candida rugosa* lipase toward esters of chiral carboxylic-acids J Org Chem 1995 60 212 217 10.1021/jo00106a036

[B31] Khuri AI Cornell JA Response Surfaces: Design and Analysis 1996 2 New York: Marcel Dekker 1 510

[B32] Li W Analysis and comparison of very large metagenomes with fast clustering and functional annotation BMC Bioinforma 2007 10 359 10.1186/1471-2105-10-359 PMC2774329 19863816

[B33] Sun S Chen J Li W Altinatas I Lin A Peltier S Stocks K Allen EE Ellisman M Grethe J Wooley J Community cyberinfrastructure for Advanced Microbial Ecology Research and Analysis: the CAMERA resource Nucleic Acids Res 2011 39 Database issue D546 D551 10.1093/nar/gkq1102 21045053 PMC3013694

[B34] Mitaku S Hirokawa T Physicochemical factors for discriminating between soluble and membrane proteins: hydrophobicity of helical segments and protein length Protein Eng 1999 12 953 957 10.1093/protein/12.11.953 10585500

[B35] Gasteiger E Hoogland C Gattiker A Duvaud S Wilkins MR Appel RD Bairoch A Walker JM Protein Identification and Analysis Tools on the ExPASy Server The Proteomics Protocols Handbook 2005 Totowa: Humana Press 571 607

[B36] Saitou N Nei M The neighbor-joining method: A new method for reconstructing phylogenetic trees Mol Biol Evol 1987 4 406 425 3447015 10.1093/oxfordjournals.molbev.a040454

[B37] Felsenstein J Confidence limits on phylogenies: an approach using the bootstrap Evolution 1985 39 783 791 10.2307/2408678 28561359

[B38] Bulow L Mosbach K The expression in E. coli of a polymeric gene coding for an esterase mimic catalyzing the hydrolysis of p-nitrophenyl esters FEBS Lett 1987 210 147 152 10.1016/0014-5793(87)81325-X 3025023

