# Supplementary material for: Isolation identification and biochemical characterization of a novel halo-tolerant lipase from the metagenome of the marine sponge Haliclona simulans
Source: Microb Cell Fact. 2012 Jun 1;11:72. doi: 10.1186/1475-2859-11-72 (PMC3544137; doi:10.1186/1475-2859-11-72)
Supplement: Additional file 1 — Supplementary files. [file 1475-2859-11-72-S1.doc]

**Supplementary files**


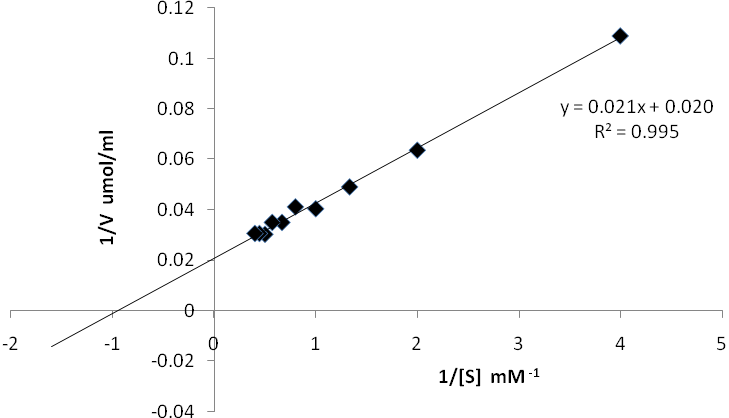


Figure 1 .Lineweaver- Burk plot for Lpc53E1 activity with *p*- nitrophenyl palmitate as substrate.

**Kinetics study by Lineweaver-Burk approach**

The kinetics of Lpc53E1 were determined by performing a Lineweaver- Burk plot with with different initial *p-* nitrophenyl palmitate concentrations (0.25- 2.5 mM). Reactions were initiated by the addition of 10nM of lipase in Tris buffer pH 8.2 and incubated at 40 ºC. The *V_max_* and K_M_ values were determined by linear regression using the following Lineweaver- Burk equation:

The K_M_ and *V_max_* value of Lipase was calculated from the slope (K_M_ /*V_max_*) and the intercept (1/*V_max_*) of Lineweaver –Burk plot.

Table 1. Experiments design matrix of response surface methodology (CCD) with experimental and predicted values of the lpc53E1 activity.

| ***Run A B C D Relative activity(%)***  ***X_1_ X_2_*** |
| --- |
| 1 -1.000 -1.000 -1.000 -1.000 76.72 73.17   2 1.000 -1.000 -1.000 -1.0 70.21 70.96  3 -1.000 1.000 -1.000 -1.000 78.25 78.95  4 1.000 1.000 -1.000 -1.000 58.23 58.46  5 -1.000 -1.000 1.000 -1.000 45.73 47.56  6 1.000 -1.000 1.000 -1.000 62.17 64.51  7 -1.000 1.000 1.000 -1.000 60.12 59.68  8 1.000 1.000 1.000 -1.000 58.21 58.36  9 -1.000 -1.000 -1.000 1.000 52.43 50.46  10 1.000 -1.000 -1.000 1.000 53.77 57.61  11 -1.000 1.000 -1.000 1.000 67.89 68.94  12 1.000 1.000 -1.000 1.000 61.47 57.82  13 -1.000 -1.000 1.000 1.000 53.67 56.84  14 1.000 -1.000 1.000 1.000 85.67 83.14  15 -1.000 1.000 1.000 1.000 84.24 81.67  16 1.000 1.000 1.000 1.000 82.76 89.71  17 -2.000 0.000 0.000 0.000 52.78 54.46  18 2.000 0.000 0.000 0.000 63.54 60.29  19 0.000 -2.000 0.000 0.000 53.86 52.71  20 0.000 2.000 0.000 0.000 65.46 65.04  21 0.000 0.000 -2.000 0.000 76.43 78.53  22 0.000 0.000 2.000 0.000 88.48 84.81  23 0.000 0.000 0.000 -2.000 62.45 62.23  24 0.000 0.000 0.000 2.000 72.23 70.87  25 0.000 0.000 0.000 0.000 100 100  26 0.000 0.000 0.000 0.000 100 100  27 0.000 0.000 0.000 0.000 100 100  28 0.000 0.000 0.000 0.000 100 100  29 0.000 0.000 0.000 0.000 100 100  30 0.000 0.000 0.000 0.000 100 100 |

A: *p*NPP (mM): -1(0.5), 0 (1.0), +1(1.5) , B: Ca^+^(mM): -1(0), 0 (5), +1(10) C: Reaction time (Min): -1 (5), 0 (15), +1 (25) and D: NaCl (M) : -1 (4), 0(5), +1(6).

| ***Source Sum of df Mean F Value p-value Squares Square Prob>F*** |
| --- |
| **Model 8717.35 14 622.67 57.73 < 0.0001*****  *A-pNPP* *50.93* *1* *50.93* *4.72* *0.0462***   *B-Ca+* *228.17* *1* *228.17* *21.15* *0.0003***   *C-Reaction time* *59.22* *1* *59.22* *5.49* *0.0333***   *D-NaCl* *111.89* *1* *111.89* *10.37* *0.0057***   *AB* *333.98* *1* *333.98* *30.96* *< 0.0001****   *AC*  *367.30* *1* *367.30* *34.05* *< 0.0001****   *AD* *87.61* *1* *87.61* *8.12* *0.0122****   *BC*  *40.26* *1* *40.26* *3.73* *0.0725****   *BD* *161.54* *1* *161.54* *14.98* *0.0015****   *CD* *1023.36* *1* *1023.36* *94.88* *< 0.0001****   *A^2^* *3 115.16* *1* *3115.16* *288.82* *< 0.0001****   *B^2^* *2899.78* *1* *2899.78* *268.86* *< 0.0001****   *C^2^* *576.19* *1* *576.19* *53.42* *< 0.0001****   *D^2^* *1917.93* *1* *1917.93* *177.82* *< 0.0001****  Residual 161.78 15 10.79  *Lack of Fit* *161.78* *10* *16.18*  *Pure Error* *0.000* *5* *0.000*  Cor Total 8879.14 29 |

Table 2. Regression analysis of enzyme assay conditions of lpc53E1 for quadratic response surface model fitting (ANOVA)

**** More significant ** significant level with P< 0.05*
